# Perspectives on cyberlearning: A case study by students, about students

**DOI:** 10.1007/s10639-022-11564-w

**Published:** 2023-01-16

**Authors:** Patricia Marybelle Davies, Reem Muteb T. Alotaishan, Hayat Khalid A. Alabdulwahed, Ali M. Fahim Khan, Rawan Mohammad Ateya, Thamer Saleh Alkhamis, Abdulaziz Abdullah A. Alodhieb

**Affiliations:** 1 College of Sciences & Human Studies, Prince Mohammad bin Fahd University, Al Khobar, Saudi Arabia; 2 College of Business Administration, Prince Mohammad bin Fahd University, Al Khobar, Saudi Arabia; 3 College of Law, Prince Mohammad bin Fahd University, Al Khobar, Saudi Arabia; 4 College of Engineering, Prince Mohammad bin Fahd University, Al Khobar, Saudi Arabia; 5 College of Computer Engineering and Science, Prince Mohammad bin Fahd University, Al Khobar, Saudi Arabia

**Keywords:** Coronavirus (covid-19), Cyberlearning, Higher education, Online learning, Saudi Arabia

## Abstract

This paper reports on a study conducted by college students at a private university in Saudi Arabia. The research examines the online learning experiences of their peers during the first wave of the coronavirus covid-19 pandemic. Many assumptions exist about online learning and its impact in higher education, but these are mainly based on the views of instructors and leaders of institutions. Hitherto, the perspectives of those meant to be beneficiaries of digital technologies have been given little consideration even though students use cyberspace for academic work and beyond. To address this silence, a group of student-researchers conducted a case study to examine students’ views of cyberlearning. The research used a qualitative analysis approach to address the following questions: (1) What were the cyberlearning experiences of students at our university during the first two semesters of lockdown? (2) What are students’ understandings of cyberlearning? (3) What are their aspirations for cyberlearning? Data were collected through an online survey administered to the entire student body at the university. Responses were received from 3574 students. The data were analysed using thematic analysis. The research participants perceive cyberlearning to be the same as online learning and see it as a viable educational option. They reported that the dominant mode of instruction in online classrooms is instructors delivering information. Respondents also highlighted the need for improved online teaching pedagogies and curbing academic dishonesty in online classrooms. Students’ aspirations for cyberlearning were clearly articulated. Respondents suggested that increasing online learning opportunities would have a positive impact on their academic progress. Through this research students demonstrate a sense of agency and provide opportunities for equity strategies at their university. The results show that serious attempts should be made to include cyberlearning as part of everyday educational activity in an attempt to increase student engagement.

## Introduction

This paper reports on research conducted by a group of six undergraduate student-researchers who are also the co-authors. The study aims to understand the online learning experiences of their peers during the unprecedented move to online education in 2020 due to the coronavirus pandemic. It examines the online learning views of students at one university in Saudi Arabia and their conceptions of cyberlearning. The research concerns a silence surrounding the implementation of online courses. It further explores how prepared students feel they and the institution are for this emerging educational approach. The student-researchers explore, analyse, and describe data collected through an empirical online survey addressing the following research questions:

1. What were the online learning experiences of students at our university during the first two semesters of lockdown?
2. What are students’ understandings of cyberlearning?
3. What are their aspirations for cyberlearning?

Personal computers first became commonplace in US educational settings in the mid-1980s (Parker & Davey, 2014). However, much of the current set-up in classrooms and libraries still relies on the views of proxy informants such as instructors, technology directors and institutional heads. Students who are meant to be the primary beneficiaries of digital technologies continue to have little or no say about which technologies are used, nor how (Sant, 2019). Students’ feedback on learning with technology, even when solicited, often goes unnoticed. One of the findings of a systematic review of online education research conducted over the past decade is that the focus has been on learner characteristics (Martin et al., 2020) instead of on teaching approaches. These authors suggest that there is a need for more research on online education to understand the perspectives of learners. What is distinctive about the present study is that it provides the first-hand accounts of college students who report on their experiences with online education at a time when they had no alternative. Conducted by students, the research exemplifies and utilises student experience to fill this critical gap in the literature.

Current demands on Higher Education Institutions (HEIs) to equip students for life in the knowledge-based, self-service, digital culture of the 21st century now make it necessary to turn the spotlight on the primary users of technology—students themselves. Students spend plenty of time in cyberspace and there is much to be garnered from them about their experiences of learning online. Often, their critiques and analyses are both constructive and non-oppositional. Researchers such as Quaglia and Fox (2018) posit that student voice helps improve institutional goals because students are engaged in a more relevant and more meaningful way with their learning environments. Furthermore, democratic notions of representation and participation as members of their academic communities now make it necessary to involve students in the online-learning debate. The covid-19 pandemic presented a crisis that has challenged the old norms and raised new questions about our educational systems. Public opinion suggest that nothing since the Second World War has had a more disruptive impact on education. For many, lockdown was a wake-up call to the reality that online learning can no longer be seen as a separate learning category. Instead, nearly all academic activities should include components in cyberspace.

Online learning can be defined as learning in an online environment using computers and the internet (Singh & Thurman, 2019). According to the National Science Foundation (NSF), cyberlearning is supporting learning through the use of networked computers and communications technologies (Borgman et al., 2008). Although similar to online learning, cyberlearning research focuses on student engagement in learning online. Inclusive of this research is understanding how people learn, how to foster learning, how to assess learning, and how to design effective learning environments. There are many variations in the scope and understanding of this new learning paradigm. Such disagreements have led to differences in opinions about the purposes of cyberlearning in education and pose problems for HEIs interested in promoting cyberlearning practices. Furthermore, this divide highlights the need for an educational public space in which to reflect collectively and discuss possible ways to expand students’ experiences of learning with technology.

The present research was designed to examine students’ views of cyberlearning and the lessons that could be learned from them. For the purposes of this paper, the terms cyberlearning and online learning are used interchangeably based on participants’ conceptions of the former (discussed further under Section 3: Results and discussion). The study is part of the first cycle in a university-wide Cyberlearning Action Research Project (CARP) being conducted to explore, document, and report on how cyberlearning is conceived, understood and appreciated by institutional stakeholders, with a view to developing educational technology policy statements for recommendation to the university’s leadership team. This first phase of CARP focuses on learners. In subsequent phases, the student-researchers will examine the views of instructors and administrators at the institution.

## Methodology

### Participants

The setting for the present study is a private university located in the Eastern Province of the Kingdom of Saudi Arabia with an undergraduate student population of just under 9000. The university has two distinct campuses, separated by gender, in adjoining locations. The six university colleges offer degrees ranging from engineering to law. Most students come from surrounding towns and cities and all are non-boarding. The survey was designed by six undergraduate student-researchers—three male and three female, sophomores to seniors—majoring in computer science, engineering, finance, and law. This multi-faceted team work jointly with the faculty supervisor facilitating the research.

The questionnaire was sent directly to students’ email accounts so the software could identify those who had not attempted the survey. A month later a reminder was sent to students who had not yet responded. In total, 3574 students completed the survey, which is around 40% of the student population at the university. Partially completed survey responses were excluded.

### Methodological approach

The research uses case study methodology, which involves the “intensive study of a single unit for the purpose of understanding a larger class of (similar) units” (Gerring, 2004, p. 342). Quantitative and qualitative data are collected but the focus is categorical data. The qualitative approach aims to understand the nature of the research problem rather than on the quantity of the characteristics that are observed at a single point in time (Strauss & Corbin, 1994). The quantitative data provide a numerical count of the numbers of students selecting particular options in the questionnaire or rating the available choices. The qualitative data, on the other hand, preserve the uniqueness of students’ perspectives on online learning and makes it possible for the action research to develop gradually.

One of the greatest strengths of qualitative research is that the dense, natural, narrative descriptions provided in the categorical data cannot be pigeon-holed nor reduced to a simple and prescriptive set of principles (Mason, 2017) as is done in quantitative research when testing a hypothesis. The focus is not on the empirical data, instead on what they mean. The data collection takes place on site, in the natural environment of students, and there are no attempts to manipulate students’ responses. The student-researchers understood the need to be ethically considerate. The British Education Research Association (BERA) (2018) Ethical Guidelines for Educational Research were followed closely. The survey was sent out by the the Student Affairs Department, which served as an agent to ensure students did not feel coerced into participating. Thus the methodological approach provided objectivity while limiting the biases of the researchers.

### Data collection

The survey instrument examined the characteristics of participants, including gender, major, college level and year of admission—to gather background information on the respondents—and explored these key areas.

- Online teaching practices: the learning technologies, instructional approaches, and assessment formats used by their instructors. In order to get an idea of teaching practices across the core curriculum, participants were asked to report on instructional practices in three subject areas—mathematics, science and humanities. Table 1 shows the survey reporting options provided. Each of these categories was used to examine the three subjects. Included as an option under each category was *Not Applicable*, which a student could choose if he or she did not take a course in that particular field.
- Conceptions of cyberlearning: this section asked students to choose from a given list of statements to demonstrate their understanding of cyberlearning. It contained a video informing participants about developments in the field.
- Aspirations for cyberlearning: this section asked for written narratives of students’ opinions of online education at the institution.
- Problems with cyberlearning: students were asked to share their perceived challenges with cyberlearning at the institution.

**Table 1:** Reporting categories in the survey

Learning Technologies	Instructional Approaches	Assessment Formats
PowerPoint or other slides	Lecture style	Comprehension
Whiteboard	Q&A	Discovery
Electronic tablet	Quiz	Research
Electronic quiz	Discussion	Construction
Break-out rooms		Collaboration
		Lab work

The validity of the questionnaire was confirmed by a panel of three experts in the field: the project facilitator who specializes in educational technology, a statistics professor, and the head of cybersecurity at the university. In preparation for the next phase of CARP, the survey also solicited participation in a student cyberlearning focus group to interrogate the survey results further and develop questions for the faculty survey, which will be sent out at a later date.

To clarify further, the aim was to examine online teaching approaches to cyberlearning—which technologies were used, and how? It did not include questions about access or learning effectiveness, nor did it aim to find out students’ preferences between traditional learning and cyberlearning. These types of questions will be discussed during the student focus group meetings to complete the data collection in Phase 1 of CARP.

### Data analysis

Descriptive statistics are used only for a visual representation of the categorical data. The bar heights represent the frequency of the categories (i.e. how many students selected that option). Utilizing the paradigm of Onwuegbuzie et al. (2016), the analysis of the qualitative data began with an intensive immersive search, which involved a process of rereading students’ narratives to gain familiarity with the responses. Persistent observation is a strategy known to provide an in-depth focus on data characteristics, thereby improving data credibility. Next, the narratives were imported into NVivo 11, which was used to further analyse the qualitative data. Descriptive open coding, a first-cycling elemental coding approach that uses a word or a short phrase to summarize data on similar topics, was employed. Then thematic analysis was used to recode emerging themes, by splitting, grouping, and eliminating codes as necessary. Simultaneous coding, the process of applying multiple codes to the same text, was also employed. This process was chosen not because of perceived ambiguity in data, but instead to capture the complex, multifaceted nature of the rich narratives provided by the participants.

The contextually relevant information helped explain trends visible in the bar charts and demonstrates the convergence of the data. Cross-checking was done in a second coding cycle using pattern coding to further consolidate the data set. This method of finding patterns or relationships among previously generated codes also served to validate the results. During each stage of the coding cycle, the results were analysed and further scrutinized by the panel of experts in an attempt to improve the trustworthiness of the findings.

## Results and discussion

### Characteristics of participants

Responses to the questions on demographics provided information on the types of students attempting the survey. The number of male respondents was 2429 (almost 68%) and over 70% of them were freshmen and sophomores. One reason for the skewness in gender might be culture. Originally limited to male students, HEIs in Saudi Arabia first enrolled women in 1962 through an off-campus program called ENTSAB (Al Alhareth et al., 2015). Recent data on gender distribution shows that over 55% of students graduating from universities in KSA are female (Statista, 2022). However, women remain reserved in sharing their views due to prevalent cultural norms, especially in the Eastern Province.

The survey respondents were distributed across all the faculties within the university. It is not unusual to have fewer upperclassmen—juniors and seniors—participating in surveys because these groups of students generally would have completed more surveys than those recently joining the university. Besides, upperclassmen are notorious for having institutional fatigue.

### Online teaching approaches

#### Learning technologies used

**Fig. 1:**
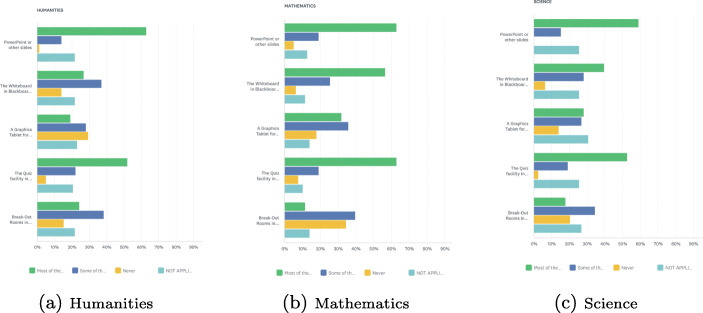
Learning technologies used by instructors

The data revealed that most instructors in the three core curriculum areas—mathematics, science and humanities—mainly use PowerPoint or other presentation slides when teaching online classes (see Fig. 1). The second most commonly used learning technology in all three subject areas was the Quiz facility in Blackboard. Respondents also reported the use of a single instructional approach, namely presentation slides, by some instructors in the humanities. This was unlike instruction in mathematics and sciences which included more varied uses of digital technologies.

#### Instructional approaches

**Fig. 2:**
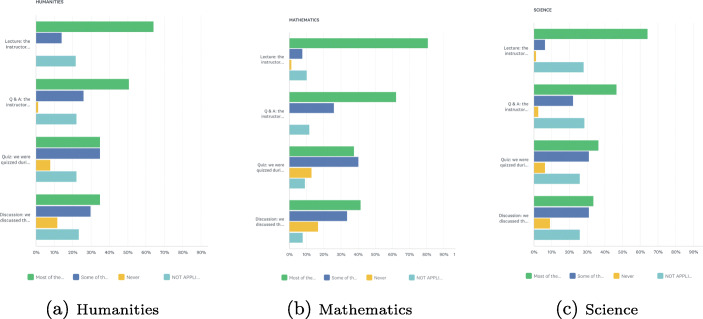
Instructional approaches used by instructors

Figure 2 shows the survey results by percentage use of instructional approaches for online courses across the three core subjects. Respondents reported that a lecture style was predominantly used, which is consistent with data in the above section showing a dominant use of presentation software. Q&A, Quizzes, and Discussions were also used frequently by mathematics instructors in addition to lectures. However, there were instructors in all three curriculum areas who never used quizzes or discussions. Lecture-based teaching aimed at transmitting information to students continues to be the primary teaching approach used in HE, even in online classrooms. Stes and Van Petegem (2014) argue that lectures can be less effective than student-focused teaching methods that involve more active learning. Thus, these results highlight an area of concern.

#### Assessment formats

**Fig. 3:**
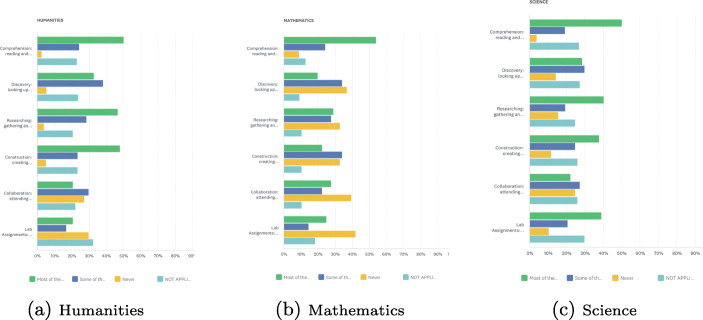
Assessment formats used by instructors

The most variation in assessment formats were reported for courses in the humanities, as shown in Fig. 3. Students reported that lecturers in these subjects provided a broad range of tasks involving collaboration, comprehension, construction, discovery, and research, compared with courses in mathematics and science. It should be noted that most of these assignments were done on paper, or using a text-editor, and had to be upload to Blackboard. Thus, the variety in humanities assessment is more due to the nature of such courses rather than because they were being delivered in cyberspace. What seems missing are the use of web-based technologies that increase social interactions and foster social learning. The majority of respondents indicated that assignments in science-related subjects predominantly involved lab work. Labs were mandatorily conducted in-person on campus.

### Conceptions of cyberlearning

To check participants’ understandings of cyberlearning, they were asked to choose all statements in the list below which they believed to be accurate.

1. Cyberlearning is the same as Virtual or Online Learning because both use electronic tools to facilitate learning.
2. Cyberlearning extends Online Learning by using technology to facilitate learning experiences that make it possible for learners to play a more active role in understanding concepts.
3. Cyberlearning is learning by searching the Internet for information.
4. Cyberlearning involves using new and developing technologies to create active learning experiences.
5. Cyberlearning investigates the future of learning with technology.

**Fig. 4:**
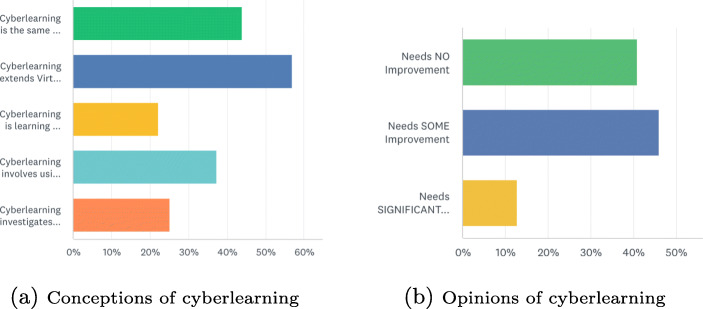
Students’ conceptions and opinions of cyberlearning

As Fig. 4a show, 43% of the respondents (1537 students) consider cyberlearning the same as online learning. Therefore, for the purposes of this report the two are used interchangeably. Even though both online learning and cyberlearning use similar digital tools, many respondents seemed unaware of the important differences between them. For example, successful online learning relies on the self-motivation of individuals, whereas cyberlearning technology is designed to motivate learners and impact how they learn. Notably, the latter is underpinned by continuing research on how to improve learning not just on making online content more interactive. Cyberlearning requires more active participation by the learner, who is driven to engage with the technology. Researchers in the field are concerned with developing technologies that help students use their minds and bodies to collaborate, think creatively and engage with new ideas in the digital world. There is also some focus on advancing computer technology in ways to expand access and equity to extend learning opportunities to all (Roschelle et al., 2017). Many cyberlearning applications now make learning fully accessible to students with disabilities.

### Aspirations for cyberlearning

The survey results showed that most participants are very excited about the prospects of formal learning being located in cyberspace. Almost 70% of the respondents (2601 students) reported being motivated or highly motivated by their online learning experiences during the first two semesters of lockdown. When asked to elaborate on their responses, some students commented that learning online was safer and more convenient than having to travel to campus during a time of uncertainty. Also, that it made them feel less anxious about participating in class discussions. Many more relished the opportunity to watch recordings of their lectures over again, something that was not previously available for in-person classes.

Figure 4b shows students’ responses to how much improvement is required by the institution in the area of online education. Some respondents added that more opportunities for online learning would be beneficial to both students and instructors. Others explained how some of the examples provided in the cyberlearning video might make learning more engaging. One respondent commented that “some lectures don’t need traditional learning approaches, and can be extremely short and to the point”. This was backed by a large number of students who indicated that online sessions force instructors to “get straight to the point”, thereby shortening class time. In effect, online learning could make it possible for classes to be located within an inquiry domain where students can experiment with simulations and data collecting sensors to learn new concepts through more participatory activities.

### Problems with cyberlearning

Along with the attributes of convenience and ease of participation respondents also mentioned the formidable challenges presented by this new genre of education. Two recurring reasons for concern given in their commentary were instructor being underprepared and academic dishonesty. Many students questioned how much training instructors had to teach online and how much experience of it. There were claims that some instructors only gave ‘audio lectures’ without any text presented while teaching in cyberspace. Designing online courses effectively is a great responsibility for instructors and can be very time consuming. Simply displaying the same content used during in-person classroom teaching on a computer screen often proves unsuccessful over the long period of a semester. Unlike the three-dimensional world of the campus classroom, the cyberclassroom is two-dimensional. So it requires different pedagogies.

Some of the challenges faced by instructors surveyed to learn about educators’ experiences during the global crisis are documented in a report by Trust and Whalen (2020). These authors highlight that many instructors are inundated and frustrated with managing continuity in teaching online due to poor internet connection, changing directives, new technologies and the unpredictability of the personal circumstances of their students. The need for instructors to be prepared to teach online also brings to bear the issue of time. Significantly higher amounts of time are needed for preparing online lessons than for in-person teaching. Also needed is some exposure to online pedagogies. The second phase of CARP will explore these issues through a questionnaire being designed for instructors.

Respondents also reported witnessing two different forms of academic dishonesty. The first involved students getting answers from a text, physical or web-based, or from another person. The second was accessing solutions through the learning platform itself; for example, when an instructor fails to put in place security measures to prevent students from retrieving answers provided for autoscoring.

Participants of the survey suggest that it is important for instructors to be aware of the different forms of online plagiarism. The limitations in identifying the authorship of digital assignments is one of the biggest barriers to adopting online education. There are now many options available for impeding students from using unsavoury methods for completing assessments, but some still find ways to get the grade they want without earning it. Academic brokering, whereby another person is paid to complete assignments for learners, or even take courses for them, has increased with online education. Mortati and Carmel (2021) allude to a *technology arms race* between instructors and students that is now under way in HEIs everywhere. They argue that the antiplagiarism tools available to instructors are not designed to tackle the ways in which academic integrity is compromised by students. One example they discuss in detail is ‘so-called’ antiplagiarism software such as TurnItIn, which they point out is not designed to detect plagiarism. Instead, what such applications evaluate are levels of originality. However, the question of whose originality often remains unanswered.

## Conclusion

This study aimed to capture the online learning experiences of university students, their understandings of cyberlearning and their aspirations for this genre of education, with a view to contributing to the development of educational technology policies. The information provided by the 3574 respondents about learning in a cyberworld revealed that the predominant pattern of online instruction across all three core subject areas was delivering information. This approach, whereby students are presented the course material, often using PowerPoint or a similar application, remains the dominant way of teaching face-to-face in HEIs. It was reported over a decade ago by Davies (2011) to be the prevalent use of technology when teaching in-person classes. Having online teaching mirror what is typically done during in-person sessions highlights the need for new online pedagogies that foster interactions with, and more independent contributions by, students in cyberclassrooms.

Such arguments are supported by recent research by Kwon et al. (2021) who contend that there is a need for instructors to think of teaching as a design science and move towards becoming designers of, and partners in, student learning in cyberspace. Also, consideration should be given to the cultural and technological changes revolutionizing 21st-century education. There is a plethora of tools that ‘so-called’ digital natives use in informal settings to develop new skills, many of which promote self-directed learning. Unfortunately, many instructors struggle with using teaching approaches that force them to relinquish control over their students.

Foregoing discussions show that students do not fully comprehend the meaning of cyberlearning. Nevertheless, they are excited about the opportunity to learn in new ways using digital tools. Over the past three decades, education has changed far less than young people have changed. Studying though the lockdown era gave students a unique perspective on what constitutes full-time learning in cyberspace. Regardless of whether or not they view cyberlearning as distinct from online learning, one message is clear—students are ready for a new genre of education that involves the cyberworld, a space where they already live, work, and play.

## Limitations and significance of the research

### Limitations

The present research does not seek to understand the challenges students faced with using the online technologies provided by the university during lockdown, or the effectiveness of online education. Nor does it examine issues relating to how and to what extent participants self-regulated their online learning activities such as is documented by Rasheed et al. (2020) in their literature review. Some of these omissions are deliberate to ensure that the focus remained on addressing the research questions. Matters dealing with access, motivation, time management, effectiveness and technology competence are no doubt significant in assessing levels of cyberlearning readiness and will be discussed by the Student Focus Group.

Also missing is information relating to levels of anxiety in students—many of whom were studying virtually for the first time—such as those addressed by (Unger and Meiran, 2020) in their recent study. It would be useful to pursue these additional lines of research, not least to understand how best to develop a gradual introduction to cyberlearning on a grand scale. Equally important are the mental issues that resulted from isolation due to university closures, which authors such as Sahu (2020) have researched. In order to ensure that the personal is not being sacrificed for the functional, further research examining these unaddressed topics is vital.

### Significance of the research

The significance of this research centres on its benefit to students. Over a decade ago, Seale (2009) critiqued the nature of student voice initiatives in HE and commented on the need for more participatory approaches. This author is concerned with the insignificant roles students often play in student voice projects. The present project went beyond using students as informants in evaluating approaches to teaching with technology during lockdown. It involved students leading the research project. Their agency raised the status of the student-researchers within the university community where they are now recognised as knowledge producers. The survey results highlight the need for pedagogical change.

A strong case for cyberlearning is that it shifts the focus of education from assessment to engagement. Having students grapple with the data produced during group activities based on embodied computing helps nurture self-esteem and develops their confidence as creators of knowledge. University students need to hone these competencies as part of their training in preparation for work. A teaching approach centered on instructors as the sole providers of information does little more than create an overdependence on knowledge being handed down. The global move to online learning during lockdown has forced educators to think deeply about alternate approaches to a 21st-century education. Therefore this study has resonance. The onus is now on HEIs to redesign education so that students are not simply preoccupied with finding ways to game the system. Without such changes, universities and colleges will continue to deliver the same product at ever-increasing prices. Online education has already helped to lower that cost for students. Prospects of cyberlearning, as it is meant to be, hold the key to delivering an improved product at an even lower cost. It is a promise of 21st-century education focused on bringing out the best in students instead of on simply measuring, often unsuccessfully, how good or bad they are.

## Data Availability

The datasets used and analysed during the current study are available from the corresponding author on reasonable request.
